# Crustacean remains from the Yuka mammoth raise questions about non-analogue freshwater communities in the Beringian region during the Pleistocene

**DOI:** 10.1038/s41598-020-57604-8

**Published:** 2020-01-21

**Authors:** Anna N. Neretina, Maria A. Gololobova, Alisa A. Neplyukhina, Anton A. Zharov, Christopher D. Rogers, David J. Horne, Albert V. Protopopov, Alexey A. Kotov

**Affiliations:** 1 A.N. Severtsov Institute of Ecology and Evolution, Leninsky Prt. 33, Moscow, 119071 Russia; 2 Faculty of Biology, M.V. Lomonosov Moscow State University, Leninskie Gory 1, building 12, Moscow, 119991 Russia; 3 Kansas Biological Survey, and The Biodiversity Institute, The University of Kansas, Higuchi Hall, 2101 Constant Avenue, Lawrence, KS 66047-3759 USA; 4 School of Geography, Queen Mary University of London, Mile End Road, London, E1 4NS UK; 5 Department of the Mammoth Fauna Studies, Academy of Sciences of the Sakha (Yakutia) Republic, Yakutsk, 677007 Russia

**Keywords:** Palaeoecology, Limnology

## Abstract

Frozen permafrost Pleistocene mammal carcasses with soft tissue remains are subject to intensive study and help elucidate the palaeoenvironment where these animals lived. Here we present an inventory of the freshwater fauna and flora found in a sediment sample from the mummified Woolly Mammoth carcass found in August 2010, from the Oyogos Yar coast near the Kondratievo River in the Laptev Sea region, Sakha (Yakutia) Republic, NE Russia. Our study demonstrates that the waterbody where the carcass was buried could be characterized as a shallow pond or lake inhabited mainly by taxa which are present in this area today, but additionally by some branchiopod crustacean taxa currently absent or unusual in the region although they exist in the arid zone of Eurasia (steppes and semi-deserts). These findings suggest that some “non-analogue” crustacean communities co-existed with the “Mammoth fauna”. Our findings raise questions about the nature of the waterbodies that existed in Beringia during the MIS3 climatic optimum when the mammoth was alive.

## Introduction

Pleistocene palaeoenvironmental studies are becoming increasingly comprehensive. Palaeo-objects are investigated by different experts, and comprehensive (multi-proxy) palaeoecological and palaeoclimatic reconstructions based on organic remains from samples, cores or outcrops are attempted^1–3^. Frozen permafrost Pleistocene mammal carcasses with soft tissue remains are especially valuable. Such specimens are subject to intensive study and help elucidate the palaeoenvironment where these animals lived^4–6^. Investigations of remains obtained from their body hair have concluded that they, unfortunately, are not contemporaneous with the animal in question and thus could not be used for palaeoenvironmental reconstructions^7^. At the same time, other approaches are possible to obtain palaeoenvironment information about where these large mammals have lived, such as analysis of gut contents, the so-called “last meal”^5,8^, or of feces^9,10^. The results of these relatively rare studies are very important as they represent “snap-shots” of the palaeoenvironment rather than a sum of collected remains integrated over possibly large time intervals, as in the case of cores and outcrop sections, where a cubic-centimeter-volume sample may in reality represent material accumulated during hundreds, if not thousands, of years. In contrast, “last meal” and feces-content studies give us information about a particular season of a particular year, and constitute rare opportunities for very precise palaeoenvironmental reconstruction.

**Figure 1:**
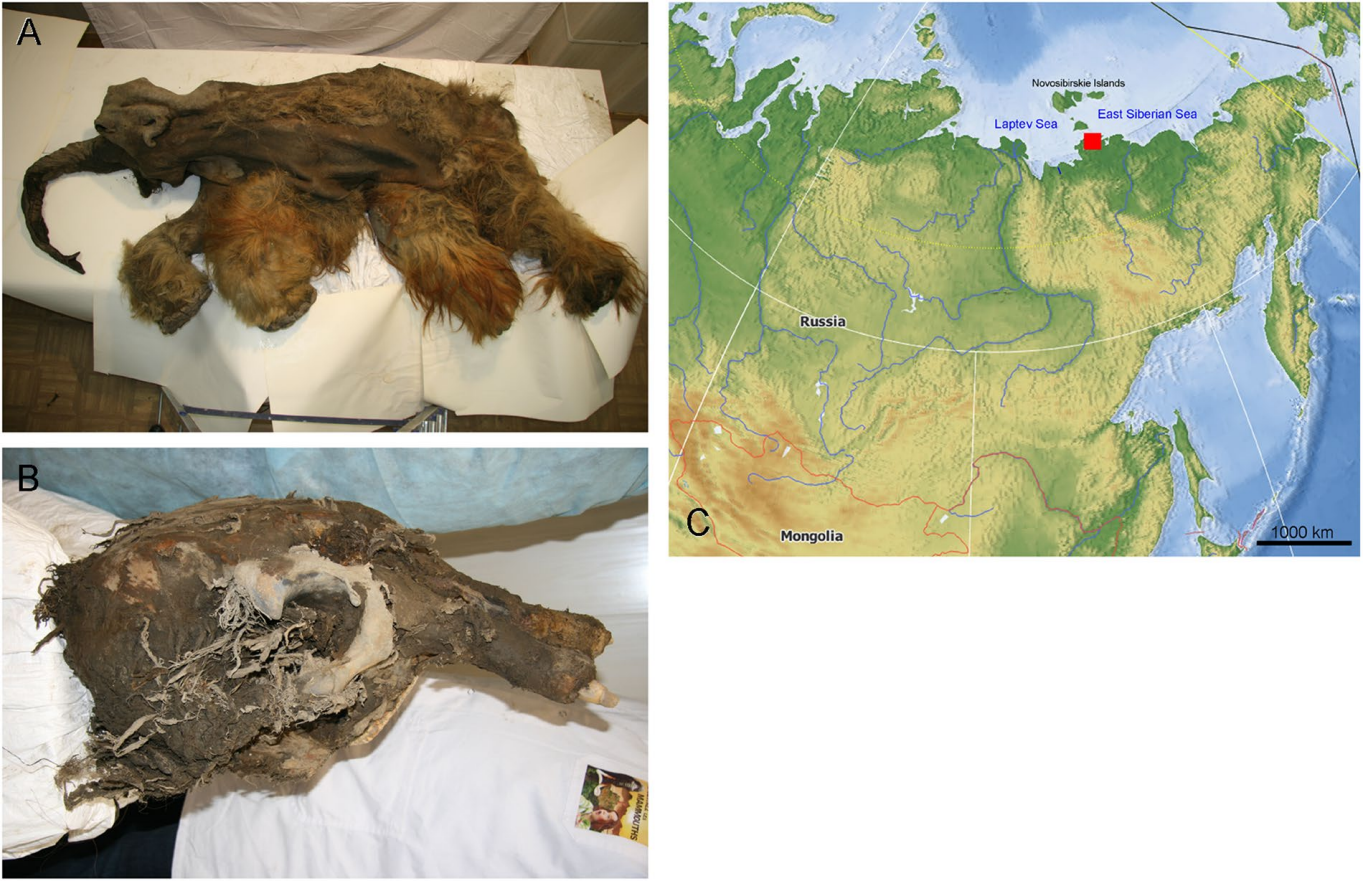
Mammoth Yuka and remains from its skull. (**A**) General view of the mammoth carcass. (**B**) Skull. (**C**) Locality where it was found on the map of Eastern Eurasia. The base map is the Marble Virtual Globe 1.5.1 “plain map” (i.e., no attributable data layers) available at https://marble.kde.org/.

Here we report on palaeoenvironmental analysis of a unique find: a mummified Woolly Mammoth carcass (Fig. 1) found in August 2010 hanging over the melting ledge in the upper third of the north-facing bank of the Kondratievo River (72.6805°N, 142.8441°E), composed of loess sediments (Oyogos Yar locality, Laptev Sea region, Sakha Republic, Russia). The juvenile (6–9 years old^11^) female was nicknamed ‘Yuka’ after the village of Yukagir, whose inhabitants discovered it. The history of this unique specimen has been documented by Kharlamova *et al*.^11^. Unfortunately it was impossible to obtain any palaeoenvironmental information from the sedimentary layers within which the carcass had been preserved, because when the workers of Sakha (Yakutia) Academy of Sciences arrived at the locality, the associated strata were already melted and could not be sampled (e.g. by coring). The bone radiocarbon date was 39.440–38.850 cal BP (GrA-53289)^12^. The specimen has been studied by different researchers for morphological and anatomical purposes, including the trunk^13^ and brain morphology^11,14^. Analysis of microfossils associated with the mammoth specimen would have been an especially interesting task but, unfortunately, only frozen sediment from the area of the skull condyles could be collected^15^ as the Yukagirs washed all the mammoth remains, including the gut, with water from a pump. Pollen and plant macrofossil analysis of these skull samples was undertaken by Rudaya *et al*.^15^.

Here we present an inventory of the freshwater fauna and flora found in the skull samples of the Yuka mammoth in order to reconstruct the waterbody type where the mammoth was preserved in the permafrost, based on taxonomic and palaeoecological analyses of crustaceans and diatoms. Our findings raise questions about the nature of the waterbodies that existed in Beringia during the MIS3 climatic optimum (ca 50–30 kyr, ages according to Andreev *et al*.^16^ and Wetterich *et al*.^17^) when the Yuka mammoth was alive.

## Results

### Invertebrates

A general list of all crustacean remains revealed in the sample is represented in Table 1.

**Table 1:** List of crustaceans revealed in the sample from skull condyles of Late Pleistocene Yuka juvenile mammoth.

Macrotaxon	Species	Amount of remains
Branchiopoda: Anostraca	*Branchinecta *? *paludosa* (O.F. Müller, 1788)	5 resting eggs
Branchiopoda: Notostraca	*Lepidurus arcticus* (Pallas, 1793)	3 mandibles, 1 maxilla, 1 telson
Branchiopoda: Anomopoda (Cladocera)	*Daphnia* (*Daphnia*) *longispina* group	17 ephippia
	*Daphnia* (*Daphnia*) *curvirostris* Eylmann, 1887	5 ephippia
	*Daphnia* (*Ctenodaphnia*) cf. *atkinsoni* Baird, 1859	2 ephippia
	*Chydorus* cf. *sphaericus* (O.F. Müller, 1776)	4 valves
Ostracoda: Podocopida: Cypridoidea	*Candona muelleri jakutica* Pietrzeniuk, 1977 (adult female)	1 valve
	*Candona* sp. (juvenile)	1 valve
	*Ilyocypris* sp. (adult, female?)	1 valve

**Figure 2:**
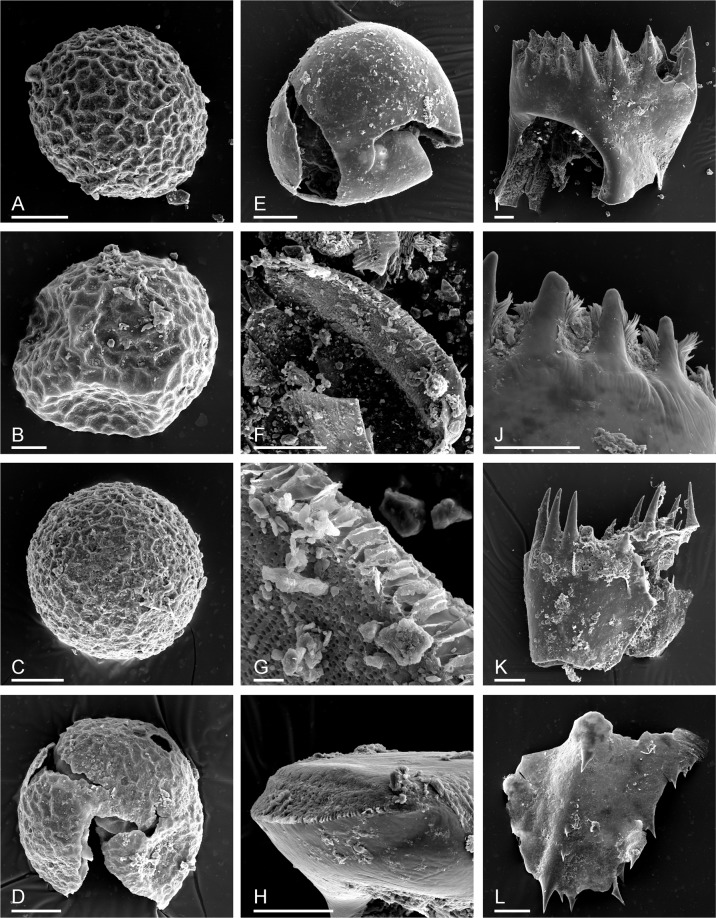
Remains of the branchiopod crustaceans from the Yuka mammoth skull. (**A**–**D**) *Branchinecta *? *paludosa*, resting eggs. (**E**) Resting egg of unknown origin. (**F**–**H**), anostracan mandible, distal view, fragment of dorsal margin and anterior view. (**I**–**L**) *Lepidurus arcticus*, distal portion of mandible, its middle portion, maxilla and telson. Scale bars 0.1 mm for (**A**–**F**,**H**–**L**); 0.01 mm for (**G**).

The anostracan eggs recovered (Fig. 2A–D) are readily identifiable as belonging to the genus *Branchinecta* Verrill, 1869 and are well within the range of variation found in *B. paludosa* O.F. Müller, 1788 egg material in the collections of DCR, as well as with previously published images^18^. These eggs are covered with a mesh of small polygons. However, as two other *Branchinecta* taxa are known from the Siberian Arctic, further specific identification is not possible. Both of the other species – *B. tolli* (Sars, 1897) and *B. minuta* Smirnov, 1948 – have not had their eggs described, nor was any material available for examination. That being said, *B. paludosa* is the most common Arctic *Branchinecta* species, also common in the Beringian region in its recent understanding^19^, and is the most widespread species in the genus, having a Holarctic distribution, occurring in seasonally dry or permafrost type shallow tundra pools.

**Figure 3:**
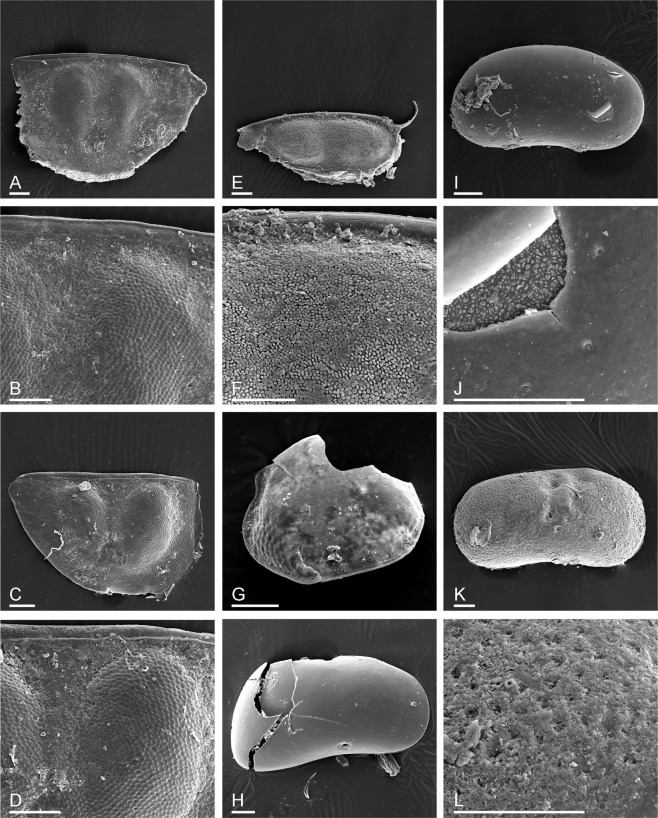
Remains of Cladocera (**A**–**G**) and Ostracoda (**H**–**L**) from the Yuka mammoth skull. (**A**,**B**) *Daphnia* (*Daphnia*) *longispina* group, ephippium and its dorsal portion. (**C**,**D**) *Daphnia* (*Daphnia*) *curvirostris*, ephippium and its dorsal portion. (**E**,**F**) *Daphnia* (*Ctenodaphnia*) *atkinsoni*, ephippium and its dorsal portion; (**G**), *Chydorus* cf. *sphaericus*, valve of female. (**H**) *Candona muelleri jakutika* adult female right valve. (**I**,**J**) *Candona* sp. juvenile right valve, with sensilla protruding from normal pores. (**K**,**L**) *Ilyocypris* sp., adult right valve (probably female) showing pitted ornamentation. Scale bars 0.1 mm.

Some resting eggs are of unknown origin (Fig. 2E). Mandibles found in the skull sample belong to the Anostraca (Fig. 3F–H), but their further identification is currently impossible as, unlike more southern taxa, Arctic and Subarctic anostracans have not been studied in this respect^20,21^.

The mandibles, maxilla and partial telson of a putative notostracan were recovered, matching the species *Lepidurus arcticus* (Pallas, 1793) quite well (Fig. 2I–L). This is the only notostracan taxon currently known to inhabit the Arctic portion of NE Eurasia, but it is also present in subarctic regions and is Holarctic in distribution^22^.

The most abundant crustacean remains are the ephippia (modified moulting exuvia of gamogenetic females containing resting eggs) of daphniids of the genus *Daphnia* O.F. Müller, 1785. Most of them (Fig. 3A,B) belong to the *D. longispina* (O.F. Müller, 1776) species group of the subgenus *Daphnia (Daphnia)* s.str. Ephippia of this group have two eggs with axes located perpendicularly to the dorsal margin, and lacking spinules on the latter^23^. Unfortunately, no identification based on ephippia of this group exists, moreover, the taxonomy of this group is confused^24^. A single exception to this rule is *D*.(*D*.) *curvirostris* Eylmann, 1887, also belonging to this group, but differing from other species in presence of small-sized spinules on its dorsal margin (but not so strong and dense as in other species of the *Daphnia (Daphnia)* group, such as *D. pulex* Leydig, 1860)^25,26^. Such ephippia are also found in our sample (Fig. 3C,D).

Two ephippia belonging to another subgenus of *Daphnia*, *D*. (*Ctenodaphnia*) Dybowski & Grochowski, 1895 (Fig. 3E,F) were found. Understanding of the taxonomy and phylogeny of the latter is clearer, and attempts to use the ephippium morphology for the taxon identification are more successful^27^. Our ephippia belong to *Daphnia* (*Ctenodaphnia*) *atkinsoni* Baird, 1859: see comments on its identification^7^.

Four valves of *Chydorus* cf. *sphaericus* (O.F. Müller, 1776) were found (Fig. 3G); their identification is based on previous works^28^.

Three specimens of ostracod crustaceans were found, all belonging to the Order Podocopida, Superfamily Cypridoidea. The first (Fig. 3H) is an adult female right valve, broken, with part of the dorsal margin missing, identified as *Candona muelleri jakutica* Pietrzeniuk, 1977; a reconstructive drawing of the broken valve produced an outline that closely matches the outline of this species as seen in published illustrations of specimens from Yakutia^29–31^. Apart from the breakage (during preparation for the SEM) the valve appears well preserved with the pseudochitinous epicuticle intact. The second specimen (Fig. 3I,J) is a juvenile of the genus *Candona* Baird, 1845, though whether it should be assigned to *C. muelleri jakutika* or to another congeneric species cannot be determined. The organic (pseudochitinous) epicuticle is preserved intact (although broken and peeling away from the mineralised calcareous procuticle in places) and there is at least one sensillum (also organic/pseudochitinous) protruding from a normal pore (Fig. 3J). Finally, the third specimen (Fig. 3K,L) is an adult right valve that can be assigned with confidence to the genus *Ilyocypris* Brady & Norman, 1889, but in the absence of soft-part preservation, identification of species of this genus is challenging. It is a close match to illustrations published as *Ilyocypris lacustris* Kaufmann, 1900^30^: (Fig. 13, images 30 and 31), an identification that we regard as questionable in view of the need for revision of several Palaearctic *Ilyocypris* species, which will require attention to phenotypic variability and small morphological features of the calcified inner lamella^32^. Most of the surface has an etched appearance, with the organic epicuticle missing, although there is a smoother patch behind the central pit where it appears to be still present.

Additionally a single head capsule of Chironomidae was found, but it was lacking the mandibles and other portions valuable for accurate species identification.

### Diatoms

A total of 86 diatom frustules were found (Table 2, S1 Fig.). Preservation of the large diatom frustules in the sample was relatively poor: they are mainly represented by fragments, which makes their precise identification difficult. Consequently, most taxa were identified to genus only. Also it was not possible to identify properly the members of the Order Fragilariales, and they were lumped into the group of “fragilarioid diatoms”. In toto, we identified 25 diatom taxa belonging to 21 genera; among them, three genera (*Aulacoseira*, *Cyclotella*, *Discostella*) belong to centric and 18 to pennate diatoms (Table 2).

**Table 2:** List of diatom taxa revealed in the sample from skull condyles of Late Pleistocene Yuka juvenile mammoth.

Taxon	Amount
**CENTRIC DIATOMS**	
Thalassiophysarales	
*Cyclotella* cf. *meneghiniana* Kützing 1844	2
*Discotella* spp.	2
Aulacoseirales	
*Aulacoseira* spp.	3
**PENNATE DIATOMS**	
Araphid diatoms	
Fragilariales	
Different species of fragilarioid diatoms	6
Tabellariales	
*Distrionella* sp.	1
*Tabellaria flocculosa* (Roth) Kützing 1844	4
**Raphid diatoms**	
Eunotiales	
*Eunotia* spp.	4
**Achnanthales**	
*Achnanthidium minutissimum* (Kützing) Czarnecki 1994 s.l.	8
*Cocconeis placentula* Ehrenberg 1838 s.l.	2
**Naviculales**	
*Cosmioneis* sp.	1
*Cymbella* sp.	1
*Encyonema* spp.	3
*Gomphoneis olivaceum* (Hornemann) P.A. Dawson ex R. Ross et P.A. Sims 1978	1
*Gomphonema* spp.	2
*Navicula cincta* (Ehrenberg) Ralfs in Pritchard 1861	3
*Navicula* cf. *salinarum* Grunow 1880	3
*Navicula* spp.	6
*Pinnularia* spp.	2
*Sellaphora* spp.	2
**Thalassiophysales**	
*Amphora copulata* (Kützing) Schoeman et R.E.M. Archibald 1986	1
*Amphora* sp.	1
**Bacillariales**	
*Grunowia solgensis* (A. Cleve) Aboal in Aboal *et al*. 2003	1
*Hantzschia amphioxys* (Ehrenberg) Grunow in Cleve et Grunow 1880	5
*Hantzschia* ssp.	3
*Nitzschia* ssp.	18
**Rhopalodiales**	
*Epithemia sorex* Kützing 1844	1
**Total**	**86**

## Discussion

Since we will never have an opportunity to obtain additional information on the precise environment where the Yuka mammoth lived, we are constrained to base our reconstruction on the single sample from the mammoth skull condyles, which is too small for an accurate quantitative analysis. Nevertheless the relatively numerous remains recovered from the sample offer evidence of the waterbody where the mammoth corpse was located shortly after (or perhaps during) its death and subsequent decomposition before finally being incorporated into the permafrost, although we cannot be certain that all of the remains are strictly autochthonous.

The taxonomic composition of the crustacean assemblage, while to a large extent characteristic of thermokarst waterbodies in the region today, also shows some unusual aspects. The large branchiopods are typical of present-day waterbodies of the region^33^. The presence of relatively numerous *Branchinecta* resting eggs is suggestive of shallow, permanent or seasonally drying pools, with high pH and dissolved calcium levels^34^. *Lepidurus arcticus* occurs in large, shallow waterbodies that dry seasonally, often co-occurring with *Branchinecta* species. Some of the other cladoceran remains are of limited palaeoecological value, mainly due to difficulties of taxonomic identification. *Chydorus* cf. *sphaericus* is a species group represented in Eurasia by at least three taxa with indistinguishable parthenogenetic females^35,36^, while gamogenetic specimens are rare. Different taxa from the *Daphnia longispina* group inhabit water bodies of different types^37^; since several taxa occur in the region this group could not be used as an indicator of abiotic factors or waterbody type. In contrast, however, two other daphniid taxa are quite remarkable. *Daphnia* (*Daphnia*) *curvirostris* occurs very rarely now in the Beringian region^38^. There are few previous reports of Pleistocene occurrences, probably because few authors have tried to identify properly the ephippia of *D*. (*Daphnia*), but we found *D. curvirostris* among the predominant taxa in the hair of a Late Pleistocene mammoth from the Allaikha River basin, Sakha Republic^26^. This taxon apparently prefers shallow, permanent or temporary waterbodies in the steppe zone and other arid regions of Eurasia, where it is very common^38^, while in the forest and tundra zones it is very rare. *Daphnia* (*Ctenodaphnia*) *atkinsoni* is currently absent in NE Asia, being known only from the Mediterranean, southern Europe and Middle Asia, mainly in arid areas^37^. Its possible relative, *D*. (*C*.) *triquetra*, is present in Kazakhstan and Mongolia^39^. Here we present the second record of this taxon from the Pleistocene Beringian zone: previously it was also found in Late Pleistocene mammoth hair from Bol’shaya Chukochya River^7^. Clearly some taxa presently occurring in the arid zone of Eurasia are now absent or rare in the Beringian region although they occurred there in the Pleistocene.

The recovered ostracod valves represent benthonic freshwater taxa consistent with a pond or small lake environment. *Candona muelleri jakutica* was originally described from Central Yakutia^31^ where it was found living in thermokarst lakes. It has subsequently been recorded living, along with other (sub-)Arctic candonine ostracod species, in low-conductivity, slightly acid to neutral, oligotrophic waters of thermokarst lakes and ponds of the Lena River Delta and the Indigirka Lowland (Northeast Siberia), and in Central and NE Yakutia (East Siberia)^29,40,41^. Species of *Candona* s.l. are benthonic crawlers or burrowers that cannot swim, while at least some *Ilyocypris* species have some swimming ability and are best regarded as nekto-benthonic. The preservation of the epicuticle and sensilla in the two *Candona* specimens suggests that they may have been only very recently dead when they were frozen and/or buried, but the etched surface of the third specimen (*Ilyocypris* sp.) seems to be indicative of post-mortem decay of the organic epicuticle, suggesting that the valve was loose on the bottom of the waterbody for some time (perhaps at least days or weeks) before freezing/burial. Co-occurrences of *C. m. jakutika* and *I. lacustris* have been recorded in MIS3 deposits of the Karginian Interstadial on the Bykovsky Peninsula (Siberian Arctic)^42^.

An accurate quantitative diatom analysis was impossible due to a scarcity of the material, and the fact that no single taxon could be regarded as dominant impedes a palaeoecological reconstruction based on diatom analysis. In addition, the identified taxa have different ecological characteristics. For example, *Eunotia* sp. and *Tabellaria flocculosa* (Roth) Kützing, 1844 are characteristic of waterbodies with low рН^43,44^ and low conductivity^45^, while *Achnanthidium minutissimum* (Kützing) Czarnecki, 1994 s.l.*, Cocconeis placentula* Ehrenberg, 1838 s.l. and *Hantzschia amphioxys* (Ehrenberg) Grunow, in Cleve et Grunow, 1880 inhabit different types of freshwater ecosystems^46^. Nowadays *T. flocculosa*, *A. minutissimum* and some other listed diatom genera are common in the polygon ponds of Arctic Siberia (e.g.^47^). Based on diatom species composition from Yuka mammoth. The only conclusion that can be drawn is that since most of the identified diatom taxa are benthic, probably the waterbody was shallow (or perhaps was a littoral zone of a larger waterbody).

The Yuka mammoth lived at the termination of the Karginsky Interstadial (MIS3 optimum). Previous palaeobotanical reconstructions led to the conclusion that the waterbody where the mammoth carcass was recovered (and where the animal had presumably died) was a small freshwater pond or shallow lake with stagnant or slowly moving water^15^. Our study demonstrates that this waterbody could be characterized as a shallow pond or lake inhabited mainly by eurytopic taxa which are present in this area today, but additionally by some taxa currently unusual in the region although they exist in the arid zone of Eurasia (steppes and semi-deserts).

Quaternary palaeoecologists have paid great attention to the reconstruction of Pleistocene Beringian climate, landscapes and terrestrial biomes^3,48,49^. Since the 1980s, a concept of “tundra-steppe” (=“mammoth steppe”) has been discussed intensively^3,50–52^. Now it is accepted that the terrestrial vegetation in Pleistocene Beringia was mosaic; in the region where the Yuka mammoth lived, “zonal tundra-steppe might have been combined with mesic-xeric meadows enriched with steppe elements”^15^. For large mammals in Pleistocene Beringia, “no-analogue communities” are reconstructed, such as the so-called “Mammoth fauna” combining taxa which are present in the region today with others that are now absent there but present in more southern regions, as well as some that are extinct^5,53^. Such non-analogue communities are also intensively discussed for higher plants^54^.

Many studies on certain model taxa of Pleistocene freshwater invertebrates and algae have been published to date (see numerous examples in^55^). However, attempts to describe the waterbodies in which these taxa occurred tend to be concentrated on certain features only, such as temperature, depth, or fish presence-absence^56–59^, rather than attempting comprehensive (bearing in mind the incompleteness of fossil records) community reconstruction.

The question of analogues between Recent and Pleistocene waterbodies has been overlooked, the assumption being that the latter could be matched with present-day types. Previous studies of Pleistocene and Holocene cladocerans from NE Asia^60,61^ were also based on the assumption that no serious changes in the ecosystems took place, only some oscillations due to climatic changes (and depth changes as a reflection of the latter), and failed to consider that “many past ecological communities were compositionally unlike modern communities”^54^.

Following recent studies^7^, we suggest the possibility that the communities in at least some Beringian Pleistocene waterbodies may also have been “non-analogue”, containing taxa recently present in the region as well as others that are apparently characteristic of waterbodies of other types, now located in much more southern and western regions and in other climatic zones (e.g. steppes and semi deserts). Examples are taxa belonging to *Daphnia* (*Ctenodaphnia*) which now are absent in the NE portion of Eurasia. Evidence of their existence in the Beringian region in the Pleistocene could be also obtained from their phylogeography: for instance, *Daphnia* (*C*.) *magna* now has a disrupted distribution area, being recorded from Eurasia and North America, but not in NE to Central Yakutia and NW Canada. It is likely that this recent biogeographic pattern has appeared as a result of the disjunction of a single pan-Beringian distribution area^62^.

We suggest, therefore, that the Pleistocene freshwater crustacean fauna could have analogies with the “Mammoth fauna”, with which some “non-analogue” crustacean communities co-existed. The timing of the collapse of the Beringian “*Ctenodaphnia* fauna” is as yet unstudied; it could be the same or different from that of the “Mammoth fauna”. A special program of studies is necessary to understand the pattern of the Beringian extinction of the branchiopod crustaceans.

## Methods

### Ethical statement

The mammoth carcass was found by local Yukagir people in their land and presented to the Yakutian Academy of Sciences. Now it is kept at Depositary of the Academy of Sciences of Sakha (Yakutia) Republic (Yakutsk), accession number ОYu-01. The stub for SEM with extracted invertebrates is deposited at the collection of Borissiak Palaeontological Institute of Russian Academy of Sciences (Moscow), collection accession number PIN RAN 5670. Slides with diatoms are deposited at the collection of the Department of Mycology and Algology, Biological Faculty of M.V. Lomonosov Moscow State University (Moscow).

### Invertebrates

The sample from the skull was thawed, dried, and sieved through a 250-μm mesh to remove coarse organic matter by Rudaya *et al*.^15^. It was screened with a stereomicroscope Leica MZ7.5, all recognizable fragments being collected using sharp needles and identified to higher taxonomic groups. Some remains were studied with a high-power Olympus CX-41 microscope. Some invertebrate remains were attached to stubs, coated with gold in a S150A Sputter Coater, and studied under a Tescan Vega TS5130MM scanning electron microscope. Individual specimens were identified using relevant literature, reference collections and personal experience.

### Diatom analysis

Material was cleaned according to Kelly *et al*.^63^ by boiling in hot hydrogen peroxide and hydrochloric acid. Six permanent slides were made using aniline-formaldehyde mountant of high refractive index. Light microscopy (LM) was performed at 1000X magnification (numerical aperture 1.40) with Leica DM2500 microscope equipped with differential interference contrast (DIC) optics and DFC 495 camera.

## Supplementary information

Supplementary information.

## Data Availability

Both mammoth carcass and specimens from hair are deposited into the collections of Governmental organizations in Russia (Depositary of the Academy of Sciences of Sakha (Yakutia) Republic, Yakutsk and Borissiak Palaeontological Institute of Russian Academy of Sciences, Moscow).
