# Supplementary material for: Crustacean remains from the Yuka mammoth raise questions about non-analogue freshwater communities in the Beringian region during the Pleistocene
Source: Sci Rep. 2020 Jan 21;10:859. doi: 10.1038/s41598-020-57604-8 (PMC6972846; doi:10.1038/s41598-020-57604-8)
Supplement: Supplementary file 1 — Supplementary information. [file 41598_2020_57604_MOESM1_ESM.pdf]

Supplementary Information to:

Crustacean remains from the Yuka mammoth raise questions about non-analogue freshwater communities in the Beringian region during the Pleistocene

Anna N. Neretina[1], Maria A. Gololobova[2], Alisa A. Neplyukhina[1], Anton A. Zharov[1], Christopher D. Rogers[3], David J. Horne[4], Albert V. Protopopov[5], Alexey A. Kotov[1]

[1] A.N. Severtsov Institute of Ecology and Evolution, Leninsky Prt. 33, Moscow 119071, Russia.

[2] Faculty of Biology, M.V. Lomonosov Moscow State University, Leninskie Gory 1, building 12, Moscow 119991, Russia.

[3] Kansas Biological Survey, and The Biodiversity Institute, The University of Kansas, Higuchi Hall, 2101 Constant Avenue, Lawrence, KS 66047-3759 USA.

[4] School of Geography, Queen Mary University of London, Mile End Road, London E1 4NS, UK.

[5] Department of the Mammoth Fauna Studies, Academy of Sciences of the Sakha (Yakutia) Republic, Yakutsk 677007, Russia.

* Correspondence and requests for material should be addressed to AAK (alexey-a-kotov@yandex.ru)

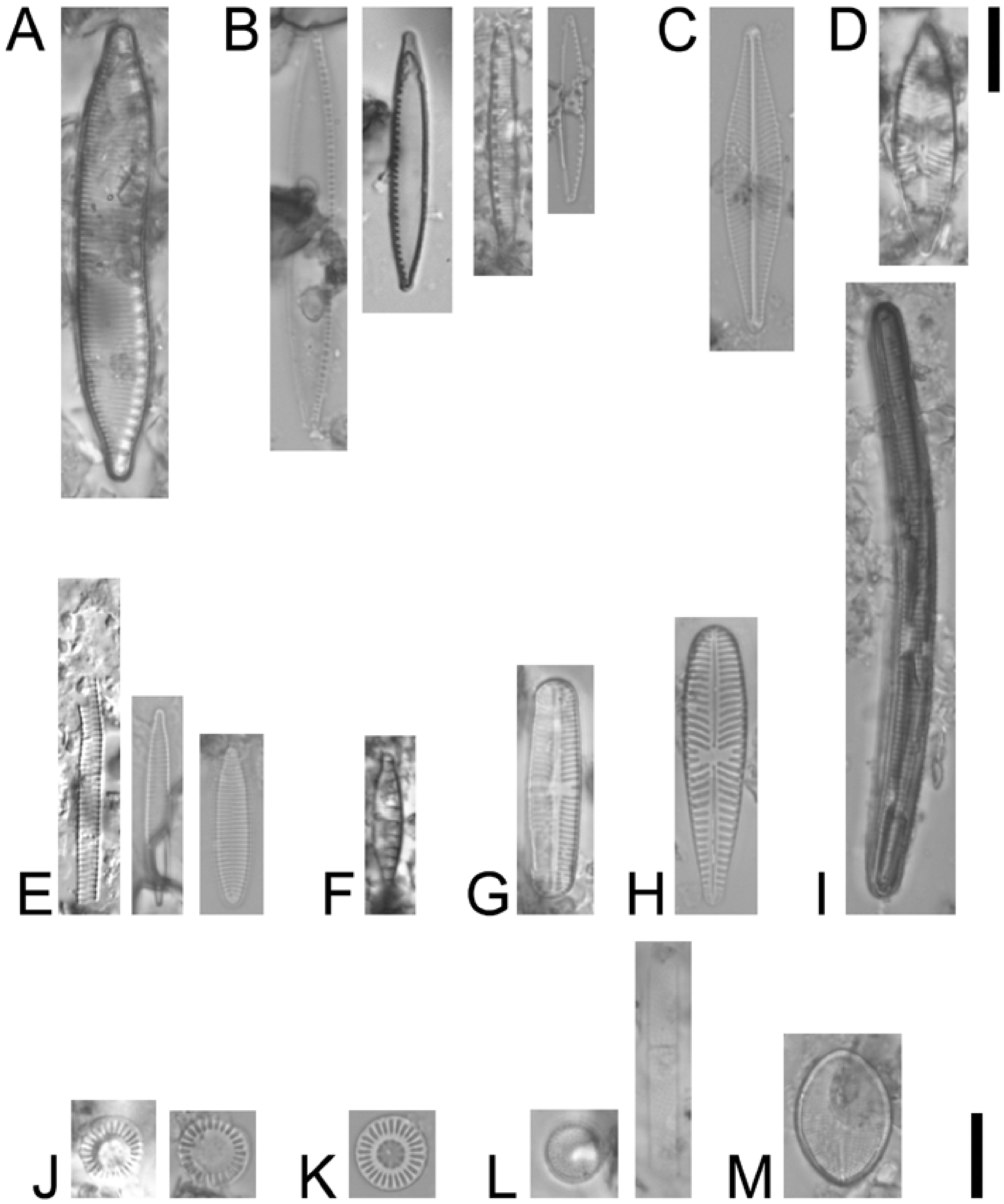

Supplementary Figure S1. Remains of diatoms from the Yuka mammoth skull. A, *Hantzschia amphioxys* (Ehrenb.) Grunow in Cleve & Grunow 1880. B, Different species of *Nitzschia* (Nitzschioid diatoms). C, *Navicula* cf. *salinarum* Grunow 1880. D, *Navicula cincta* (Ehrenb.) Ralfs in Pritchard 1861. E, Different species of Fragilarioid diatoms. F, *Grunowia solgensis* (A.Cleve) Aboal in Aboal et al. 2003. G, *Sallaphora* sp. H, *Gomphoneis olivaceum* (Hornem.) P.A.Dawson ex R.Ross et P.A.Sims 1978. I, *Eunotia* sp. J, *Cyclotella* cf. *meneghiniana* Kütz. 1844. K, *Discotella* sp. L, *Aulacoseira* sp. M, *Cocconeis placentula* Ehrenb. 1838 s. l. Scale bar represents 0.01 mm.
